# Effects of Levonorgestrel-Releasing Intrauterine Device Therapy on Ovarian Reserve in Menorrhagia

**DOI:** 10.7759/cureus.31721

**Published:** 2022-11-21

**Authors:** Soner Gök, Erkan Alataş

**Affiliations:** 1 Obstetrics and Gynecology, Pamukkale University, Denizli, TUR

**Keywords:** therapy, ovarian reserve, menorrhagia, levonorgestrel-releasing intrauterine device, anti-müllerian hormone

## Abstract

Objective

This study aimed to investigate the effects of levonorgestrel-releasing intrauterine device (LNG-IUD) treatment on ovarian reserve in women of reproductive age diagnosed with menorrhagia.

Methods

This was a prospective controlled trial involving 50 women with menorrhagia and a control group comprising age-matched 50 healthy women. Women who satisfied the LNG group criteria underwent an endometrial pipelle biopsy and LNG-IUD insertion. Ovarian reserve tests were performed prior to and six months after LNG-IUD insertion in the LNG group cases.

Results

Follicle-stimulating hormone (FSH), luteinizing hormone (LH), estradiol (E2), anti-Müllerian hormone (AMH), endometrial thickness (ET), total antral follicle count (AFC), and mean ovarian volume values before LNG-IUD insertion did not differ between the LNG and control groups. When the final measurements were compared, FSH, AMH, total AFC, and average ovarian volume increased (p=0.05, 0.046, 0.022, and 0.022, respectively), E2 and ET decreased (p=0.034 and 0.001, respectively) in the LNG group, while LH did not differ significantly between the groups (p=0.71).

Conclusion

We observed that LNG-IUD use effectively improves fertility capacity. In this study, LNG-IUD use in reproductive-age women diagnosed with menorrhagia decreased E2 levels, did not change LH levels, and increased FSH, AFC, and AMH levels.

## Introduction

Menorrhagia, also called heavy menstrual bleeding, is defined as excessive menstrual bleeding (≥80 mL) that impairs a woman’s physical, social, and emotional quality of life. It may occur alone or in combination with other symptoms [1]. Menorrhagia causes iron deficiency anemia in 60-70% of cases, and some of these women have to go undergo surgery within five years [2]. For women of reproductive age, menorrhagia is among the most common reasons for visiting a gynecology clinic [3]. Despite the widespread use of oral progesterone in the treatment of menorrhagia for two decades, patient compliance has been limited due to the reluctance of many women to take oral drugs for prolonged periods as well as their side effects [4].

One of the most significant discoveries in gynecology in recent years is the levonorgestrel-releasing intrauterine device (LNG-IUD). This plastic T-shaped device's vertical stem contains 52 mg of levonorgestrel in it, which is released daily for five years at a dose of 20 μgr. The daily dose of levonorgestrel causes decidualization of the endometrial stroma, atrophy of the endometrial glands, a surface papillary pattern, and a stromal inflammatory infiltrate [5]. Although it was originally produced as a contraceptive device, it is now widely used as menorrhagia and hormone replacement therapy [6,7]. However, there is a paucity of studies on the effects of LNG-IUD, which is increasingly used in women of reproductive age due to menorrhagia, on the ovarian reserve of these patients. 

Ovarian reserve measures the number and quality of oocytes capable of folliculogenesis and steroidogenesis within the ovarian tissue [8]. Anti-Müllerian hormone (AMH) and antral follicle count (AFC), which are substitutes for the actual ovarian reserve, are the two most frequently used markers of ovarian reserve [9,10]. Based on histological findings, AMH and AFC reflect the size of the primordial follicle pool [9] and correlate with the natural timing of menopause [11,12]. Serum levels of follicle-stimulating hormone (FSH) and luteinizing hormone (LH), which are gonadotropic hormones that govern the menstrual cycle, also provide information about ovarian reserve [13,14]. AFC, together with ovarian volume measurement and Doppler ovarian blood flow indices, are critical measures of ovarian reserve. Ovarian volume is also a good ovarian reserve marker [15].

In this context, the main purpose of our study was to determine whether the LNG-IUD, which is increasingly used in the treatment of menorrhagia in women of reproductive age, has an effect on the ovarian reserve of these patients and to compare it with a healthy control group.

## Materials and methods

Institutional review board approval

This case-controlled prospective study was conducted between November 2020 and November 2021 in accordance with the principles of the Declaration of Helsinki. All participants provided written and informed consent prior to participating in the study. Ethical approval was obtained from the Pamukkale University Clinical Research Ethics Committee (13.10.2020: 19).

Study participants and design

A total of 158 Turkish women of reproductive who were consecutively admitted to the department of gynecology at the study center were deemed eligible for the study. Among those, eight women with pelvic pathologies (cervical and/or endometrial polyp, myoma, mass or cyst in the ovary), five women with systemic diseases, three women who had acute infections, six women who regularly used combination oral contraceptive (COC), three women who used anticoagulants, two women who had endometrial premalignant lesions detected on the endometrial pipelle biopsy, 19 women without regular menstrual cycles at the six-month follow-up after LNG-IUD insertion, four women who did not attend the follow-up examination after six cycles, and five women who refused to participate were excluded. A total of 50 women who had regular menstrual cycles (occurring in 21-35 days) and who experienced heavy menstrual bleeding (menorrhagia) were assigned to the LNG group. A total of 50 women who had regular menstrual cycles (occurring in 21-35 days) without menorrhagia or any known medical disease were assigned to the control group.

The participants were instructed to complete a guided self-assessment questionnaire that documented their demographic features and clinical characteristics about menstruation. Height and weight were measured, and body mass index (BMI, in kg/m^2^) was calculated.

Study plan and interventions

An endometrial pipelle biopsy was performed in women who presented with menorrhagia. Women with premalignant or malignant pathology on the biopsy were excluded from the study, while non-premalignant or non-malignant cases were called for control on the third to the fifth day of the cycle. Venous blood samples were obtained after a 12-hour fast on the third to the fifth day of the cycle. Serum FSH, LH, E2, and AMH levels, and hemoglobin and hematocrit levels were evaluated in the blood samples. All transvaginal USGs were conducted by the same investigator in all instances on the day of the blood sampling. Endometrial thickness (ET), AFC, and volume of both ovaries were assessed using transvaginal USG. For ET, double-wall measurements were performed at the thickest point in the longitudinal segment. To compute the follicle sizes, the average of the measurements of the diameters in three planes was calculated, and the follicles with a diameter of 2-10 mm were considered antral. Ovarian volumes were determined using the formula D1 × D2 × D3 × 0.52 after measuring the diameters in three vertical planes for each ovary. LNG-IUD was inserted into the endometrial cavity on the sixth to 10th day of the cycle in all cases in the LNG group. After six cycles (approximately six months), serum FSH, LH, E2, and AMH levels, and hemoglobin and hematocrit levels were re-evaluated after a 12-hour fast on the third to the fifth day of the menstrual cycle, and a transvaginal USG was performed again by the same investigator. ET, AFC, and both ovarian volume measurements of cases in which an LNG-IUD was detected in the endometrial cavity on transvaginal USG were repeated, and all values were recorded.

Venous blood samples were taken from healthy volunteer women in the control group on the third to the fifth day of the cycle after a 12-hour fast. Serum levels of FSH, LH, E2, and AMH, and hemoglobin and hematocrit levels were evaluated. The same investigator performed transvaginal USG in all cases on the day the serum samples were collected. Transvaginal USG was used to determine the ET, AFC, and volume of both ovaries. As in the LNG group, after six cycles (approximately six months), serum FSH, LH, E2, and AMH levels, and hemoglobin and hematocrit levels were re-evaluated after a 12-hour fast on the third to the fifth day of the menstrual cycle, and the transvaginal USG was repeated by the same investigator. All measurements of ET, AFC, and both ovarian volumes were repeated on the transvaginal USG, and all values were recorded. Since the control group consisted of healthy women without menorrhagia, no endometrial biopsy was performed and LNG-IUD was not inserted.

Statistical analysis

All variables were analyzed descriptively. The mean, standard deviation (SD), and median were used to express descriptive statistics for numerical variables, while number and percentage were used to express categorical variables. SPSS Statistics for Windows version 25.0 (IBM Corp., Armonk, NY) was used to conduct the statistical analyses. The Kolmogorov-Smirnov test was used to determine whether the data were normally distributed. Student’s t-test was used to compare features that were normally distributed in two independent groups, and the Mann-Whitney U test was used to examine features that were not normally distributed in two independent groups. The Friedman test and the corrected Shapiro-Wilk test were used to investigate characteristics that did not exhibit a normal distribution on repeated occasions. Repeated measurements were compared between the control and study groups using the two-way analysis of variance (ANOVA).

## Results

There were no statistically significant intergroup differences in demographic characteristics such as age, BMI, parity, or gravidity (p=0.870, 0.343, 0.790, and 0.896, respectively) (Table 1). While there was no significant intergroup difference in menstrual cycle length (p=0.532), menstrual cycle duration and menstrual bleeding were significantly higher in the LNG group (p<0.001) (Table 1).

**Table 1:** Comparison of demographic, reproductive, and sonographic characteristics between control and LNG groups *Statistically significant at p<0.05 SD: standard deviation; Hgb: hemoglobin; Htc: hematocrit; FSH: follicle-stimulating hormone; LH: luteinizing hormone; E2: estradiol; AMH: anti-Müllerian hormone; AFC: antral follicle count

Variables		Control group (n=50), mean ± SD	LNG group (n=50), mean ± SD	P-value
Age (year)		35.96 ± 1.7	36.10 ± 1.8	0.870
Body mass index (kg/m^2^)		26.78 ± 2.6	27.53 ± 2.5	0.343
Parity		2.30 ± 0.70	1.96 ± 0.78	0.790
Gravida		2.50 ± 0.83	2.22 ± 0.89	0.896
Menstrual cycle length (days)		27.88 ± 1.95	28.12 ± 1.87	0.532
Menstrual cycle duration (days)		5.10 ± 0.91	6.26 ± 0.94	<0.001*
Menstrual bleeding (pads/day)		5.14 ± 0.90	8.48 ± 1.1	<0.001*
Hgb (g/dl)	First level	12.64 ± 0.84	11.18 ± 0.96	<0.001*
	Final level	12.44 ± 0.94	12.83 ± 0.85	0.035*
Htc (%)	First level	37.59 ± 2.70	33.41 ± 2.98	<0.001*
	Final level	37.13 ± 2.96	38.30 ± 2.79	0.042*
FSH (mIU/ml)	First level	7.46 ± 1.40	7.82 ± 1.29	0.422
	Final level	7.64 ± 1.50	8.34 ± 1.66	0.05*
LH (mIU/ml)	First level	3.70 ± 1.15	3.98 ± 1.04	0.234
	Final level	3.64 ± 1.19	3.82 ± 1.22	0.710
E2 (ng/L)	First level	44.96 ± 11.31	44.52 ± 12.37	0.150
	Final level	46.18 ± 8.89	41.28 ± 12.42	0.034*
AMH (ng/ml)	First level	3.02 ± 3.25	3.25 ± 1.60	0.439
	Final level	2.99 ± 3.64	3.64 ± 1.86	0.046*
Endometrial thickness (mm)	First measure	7.44 ± 1.98	8.02 ± 2.05	0.226
	Final measure	7.32 ± 1.38	5.06 ± 1.20	<0.001*
Total AFC	First measure	8.86 ± 1.64	9.02 ± 1.64	0.208
	Final measure	8.78 ± 9.54	9.54 ± 1.83	0.022*
Average ovarian volume (mm^3^)	First measure	5.93 ± 6.20	6.20 ± 1.28	0.208
	Final measure	6.01 ± 6.56	6.56 ± 1.29	0.022*

While the first levels of hemoglobin and hematocrit were significantly higher in the control group (p<0.001), the final levels were found to be significantly higher in the LNG group (p=0.035 and 0.042, respectively) (Table 1). There were no significant intergroup differences in the first measurements of FSH, LH, E2, AMH, ET, total AFC, or average ovarian volume (p=0.422, 0.234, 0.150, 0.439, 0.226, 0.208, and 0.208, respectively) (Table 1). When the final measurements were compared, FSH, AMH, total AFC, and average ovarian volume increased (p=0.05, 0.046, 0.022, and 0.022, respectively) and E2 and ET decreased (p=0.034 and 0.001, respectively) in the LNG group, while LH did not differ significantly between the groups (p=0.71) (Table 1).

The Wilcoxon signed-rank test was used to determine the differences between the first and final measurements (Table 2). There were no statistically significant differences between the first and final measurements in the control group (p>0.05) (Table 2). When the first and final measurements in the LNG group were compared, FSH, AMH, total AFC, and average ovarian volume increased (p=0.012, 0.001, 0.002, and 0.001, respectively), LH did not change (p=0.408), and E2 and ET decreased (p=0.001 and 0.001, respectively) (Table 2).

**Table 2:** Comparison between the first and final values of FSH, LH, E2, AMH, and sonographic characteristics *Statistically significant at p<0.05 SD: standard deviation; LNGG: levonorgestrel group; CG: control group; FSH: follicle-stimulating hormone; LH: luteinizing hormone; E2: estradiol; AMH: anti-Müllerian hormone; AFC: antral follicle count

Variables		First value, mean ± SD	Final value, mean ± SD	P-value
FSH (mIU/ml)	LNGG	7.82 ± 1.29	8.34 ± 1.66	0.012*
	CG	7.46 ± 1.40	7.64 ± 1.51	0.353
LH (mIU/ml)	LNGG	3.98 ± 1.04	3.82 ± 1.22	0.408
	CG	3.70 ± 1.15	3.64 ± 1.19	0.787
E2 (ng/L)	LNGG	44.52 ± 12.37	41.28 ± 12.42	<0.001*
	CG	44.96 ± 11.31	46.18 ± 11.79	0.146
AMH (ng/ml)	LNGG	3.25 ± 1.60	3.64 ± 1.86	<0.001*
	CG	3.02 ± 1.61	2.99 ± 1.67	0.409
Endometrial thickness (mm)	LNGG	8.02 ± 2.05	5.06 ± 1.20	<0.001*
	CG	7.44 ± 1.98	7.32 ± 1.38	0.532
Total AFC	LNGG	9.02 ± 1.64	9.54 ± 1.83	0.002*
	CG	8.86 ± 1.64	8.78 ± 1.69	0.511
Average ovarian volume	LNGG	6.20 ± 1.28	6.56 ± 1.29	<0.001*
	CG	5.93 ± 1.12	6.01 ± 1.28	0.232

## Discussion

Menorrhagia is seen in a significant proportion of gynecology patients and affects their physical, social, and emotional well-being. In recent years, LNG-IUD, which has been used in the treatment of menorrhagia, has become an alternative to hysterectomy because of its effectiveness in women with excessive menstrual bleeding [7]. Progestins are substances that act in a variety of ways on progesterone receptors, including anovulation, a relatively hypoestrogenic state, decreased FSH and LH secretion, and amenorrhea that prevents menorrhagia. Moreover, they have antiestrogenic effects, causing endometrial pseudodecidualization, inhibiting inflammatory response, provoking apoptosis of endometriotic cells, reducing oxidative stress, inhibiting angiogenesis, and suppressing the expression of matrix metalloproteinases [16,17]. LNG-IUD can lead to reduced endometrial cell proliferation and increased apoptosis, which also causes endometrial glandular atrophy and decidual transformation of the stroma. A 70-90% decrease in menstrual blood loss is seen after the first year of use. Indeed, studies have shown that LNG-IUD is an effective treatment option for heavy menstrual bleeding [18,19]. LNG-IUD provides an opportunity for patients with menorrhagia to preserve their reproductive function and eliminate their surgical risks and costs [20]. We also used LNG-IUD as the menorrhagia treatment method in our study, and we found a significant increase in hemoglobin and hematocrit parameters in the sixth month of LNG-IUD treatment.

Our study investigated the effects of the LNG-IUD on the ovarian reserve of women with menorrhagia of reproductive age. To that end, we evaluated FSH, LH, E2, AMH, ET, total AFC, and average ovarian volume values prior to and six months after LNG-IUD insertion. While many studies have examined the effect of LNG-IUD on uterine bleeding in the literature, very few have investigated its effects on ovarian reserve. Studies on the effects of LNG-IUD on ovarian function generally compare contraceptive methods [21,22].

Menorrhagia is generally a leading reason for women of reproductive age to consult a gynecologist. The demographic characteristics of the patients in the LNG group were as follows - mean age: 36.1 years; BMI: 27.5 kg/m^2^; and parity: 1.96. There was no statistically significant intergroup difference in demographic characteristics. This result shows that the groups had similar demographic features, and the comparative analyses were consistent.

AMH is a dimeric glycoprotein that is secreted from the granulosa cells surrounding the preantral and antral follicles [23]. In addition to its functional role in the ovary, AMH affects the number of preantral follicles that make up the oocyte pool, making serum AMH level a marker of ovarian reserve [24]. According to some authors, gonadotropins, particularly FSH, prevent the production of serum AMH. On the other hand, the stimulating effect of FSH on AMH expression in normal and polycystic ovaries has been described [25]. This controversial point could be reconciled by recent findings that E2 inhibits AMH expression mediated via estrogen receptor ß [26]. In small antral follicles, FSH could directly stimulate AMH, but by increasing E2 production in larger follicles, FSH may inhibit AMH expression through the negative feedback of E2 [26]. A study by Landersoe et al. [21] that compared AMH and AFC among contraceptive users noted approximately 20% higher levels of AMH and AFC among LNG-IUS users than among combination oral contraceptive (COC) users, but there was no significant difference between COC users and progestin-only pill or contraceptive vaginal ring users. They also noted that in fully adjusted models, AMH levels were 17.1% lower among women using LNG-IUS.

Hariton et al. [22] compared AMH among contraceptive users and women not on contraceptives and found that AMH levels were 7% lower in women with hormonal IUD, 15% lower in those taking the progestin-only pill, 22% lower in vaginal ring users, 23% lower in implant users, and 24% lower in those taking COC. They also noted that the AMH level in women using copper IUDs was not significantly different from that in women not using contraceptives. In the current study, we found a decrease in E2, an increase in FSH and AMH, and no change in LH at six months after versus prior to LNG-IUD insertion in the setting of menorrhagia. We obtained the same results in the comparison of six-month values between cases and controls. Our results contrast with those of the above studies among contraceptive users. We hypothesized that this may be due to the suppression of E2 production by low-dose progestins secreted from the LNG-IUD, which may have caused a temporary increase in FSH secretion at the beginning, resulting in an increase in AMH secretion in the early period.

AFC, an ultrasonographic ovarian reserve evaluation method, is the ovarian reserve test with the highest predictive value for predicting ovarian response with or without AMH [27]. Therefore, here we evaluated the AFC and mean ovarian volumes in all cases. According to the results of our study, there was a significant increase in AFC and mean ovarian volume six months after vs. prior to LNG-IUD insertion.

The potent ethinylestradiol component of COC and the contraceptive vaginal ring, in conjunction with a high progestin dose, significantly suppresses the hypothalamus-pituitary to produce the contraceptive effects. This leads to decreased secretion of FSH and blocking of the LH surge, which results in the inhibition of E2 production, follicular growth, and ovulation [28]. LNG-IUD usually causes amenorrhoea by inhibiting endometrial growth and causing an atrophic endometrium [5,29]. Ovulation inhibition by LNG-IUD use appears partial and dose-dependent [29]. In addition, amenorrhoea may occur despite ovulation because of an atrophic endometrium [29]. This leads to thinning of the functional layer of the endometrium. The thinning of the ET observed in our investigation was similar to the results of previous studies [30].

The following study limitations were identified: (1) this was a single-center study, (2) a group with irregular menstrual cycles after LNG-IUD insertion was not included, (3) cases with leiomyoma, endometrioma, and polycystic ovary syndrome (PCOS) were not evaluated (only primary menorrhagia was evaluated), and (4) cases were evaluated after only six months.

## Conclusions

We observed that LNG-IUD use effectively improves fertility capacity. In this study, LNG-IUD use in reproductive-age women diagnosed with menorrhagia decreased E2 levels, did not change LH levels, and increased FSH, AFC, and AMH levels. We believe that these effects on ovarian reserve may be the systemic reflection of the effects of short-term intrauterine progestin administration on the endometrium, and/or the elimination of the stress caused by anemia due to increased hemoglobin and hematocrit parameters. Based on the treatment outcomes reported here, women with menorrhagia of reproductive age should be able to make a well-informed decision about the effect of treatment on fertility. Further studies are required to confirm our findings.

## References

[1] Matthews ML (2015). Abnormal uterine bleeding in reproductive-aged women. Obstet Gynecol Clin North Am.

[2] Deneris A (2016). PALM-COEIN nomenclature for abnormal uterine bleeding. J Midwifery Womens Health.

[3] Nicholson WK, Ellison SA, Grason H, Powe NR (2001). Patterns of ambulatory care use for gynecologic conditions: a national study. Am J Obstet Gynecol.

[4] Committee on Practice Bulletins—Gynecology (2012). Practice bulletin no. 128: diagnosis of abnormal uterine bleeding in reproductive-aged women. Obstet Gynecol.

[5] Sheppard BL (1987). Endometrial morphological changes in IUD users: a review. Contraception.

[6] Inki P (2007). Long-term use of the levonorgestrel-releasing intrauterine system. Contraception.

[7] Roberts TE, Tsourapas A, Middleton LJ (2011). Hysterectomy, endometrial ablation, and levonorgestrel releasing intrauterine system (Mirena) for treatment of heavy menstrual bleeding: cost effectiveness analysis. BMJ.

[8] Cohen PE, Holloway JK (2010). Predicting gene networks in human oocyte meiosis. Biol Reprod.

[9] Hansen KR, Hodnett GM, Knowlton N, Craig LB (2011). Correlation of ovarian reserve tests with histologically determined primordial follicle number. Fertil Steril.

[10] La Marca A, Sighinolfi G, Radi D (2010). Anti-Mullerian hormone (AMH) as a predictive marker in assisted reproductive technology (ART). Hum Reprod Update.

[11] Nair S, Slaughter JC, Terry JG (2015). Anti-mullerian hormone (AMH) is associated with natural menopause in a population-based sample: the CARDIA Women's Study. Maturitas.

[12] Depmann M, Eijkemans MJ, Broer SL, Scheffer GJ, van Rooij IA, Laven JS, Broekmans FJ (2016). Does anti-Müllerian hormone predict menopause in the general population? Results of a prospective ongoing cohort study. Hum Reprod.

[13] Dinh A, Sriprasert I, Williams AR, Archer DF (2015). A review of the endometrial histologic effects of progestins and progesterone receptor modulators in reproductive age women. Contraception.

[14] Nelson LR, Bulun SE (2001). Estrogen production and action. J Am Acad Dermatol.

[15] Broekmans FJ, Kwee J, Hendriks DJ, Mol BW, Lambalk CB (2006). A systematic review of tests predicting ovarian reserve and IVF outcome. Hum Reprod Update.

[16] Reis FM, Coutinho LM, Vannuccini S, Batteux F, Chapron C, Petraglia F (2020). Progesterone receptor ligands for the treatment of endometriosis: the mechanisms behind therapeutic success and failure. Hum Reprod Update.

[17] Barra F, Scala C, Ferrero S (2018). Current understanding on pharmacokinetics, clinical efficacy and safety of progestins for treating pain associated to endometriosis. Expert Opin Drug Metab Toxicol.

[18] Endrikat J, Shapiro H, Lukkari-Lax E, Kunz M, Schmidt W, Fortier M (2009). A Canadian, multicentre study comparing the efficacy of a levonorgestrel-releasing intrauterine system to an oral contraceptive in women with idiopathic menorrhagia. J Obstet Gynaecol Can.

[19] Endrikat J, Vilos G, Muysers C, Fortier M, Solomayer E, Lukkari-Lax E (2012). The levonorgestrel-releasing intrauterine system provides a reliable, long-term treatment option for women with idiopathic menorrhagia. Arch Gynecol Obstet.

[20] Lethaby A, Hussain M, Rishworth JR, Rees MC (2015). Progesterone or progestogen-releasing intrauterine systems for heavy menstrual bleeding. Cochrane Database Syst Rev.

[21] Landersoe SK, Forman JL, Birch Petersen K (2020). Ovarian reserve markers in women using various hormonal contraceptives. Eur J Contracept Reprod Health Care.

[22] Hariton E, Shirazi TN, Douglas NC, Hershlag A, Briggs SF (2021). Anti-Müllerian hormone levels among contraceptive users: evidence from a cross-sectional cohort of 27,125 individuals. Am J Obstet Gynecol.

[23] Jeppesen JV, Anderson RA, Kelsey TW (2013). Which follicles make the most anti-Mullerian hormone in humans? Evidence for an abrupt decline in AMH production at the time of follicle selection. Mol Hum Reprod.

[24] Lemos NA, Arbo E, Scalco R, Weiler E, Rosa V, Cunha-Filho JS (2008). Decreased anti-Müllerian hormone and altered ovarian follicular cohort in infertile patients with mild/minimal endometriosis. Fertil Steril.

[25] Panidis D, Katsikis I, Karkanaki A, Piouka A, Armeni AK, Georgopoulos NA (2011). Serum anti-Müllerian hormone (AMH) levels are differentially modulated by both serum gonadotropins and not only by serum follicle stimulating hormone (FSH) levels. Med Hypotheses.

[26] Grynberg M, Pierre A, Rey R (2012). Differential regulation of ovarian anti-müllerian hormone (AMH) by estradiol through α- and β-estrogen receptors. J Clin Endocrinol Metab.

[27] Domingues TS, Rocha AM, Serafini PC (2010). Tests for ovarian reserve: reliability and utility. Curr Opin Obstet Gynecol.

[28] Vandever MA, Kuehl TJ, Sulak PJ, Witt I, Coffee A, Wincek TJ, Reape KZ (2008). Evaluation of pituitary-ovarian axis suppression with three oral contraceptive regimens. Contraception.

[29] Apter D, Gemzell-Danielsson K, Hauck B, Rosen K, Zurth C (2014). Pharmacokinetics of two low-dose levonorgestrel-releasing intrauterine systems and effects on ovulation rate and cervical function: pooled analyses of phase II and III studies. Fertil Steril.

[30] Mittermeier T, Farrant C, Wise MR (2020). Levonorgestrel-releasing intrauterine system for endometrial hyperplasia. Cochrane Database Syst Rev.

